# A Large Animal Model for *CNGB1* Autosomal Recessive Retinitis Pigmentosa

**DOI:** 10.1371/journal.pone.0072229

**Published:** 2013-08-19

**Authors:** Paige A. Winkler, Kari J. Ekenstedt, Laurence M. Occelli, Anton V. Frattaroli, Joshua T. Bartoe, Patrick J. Venta, Simon M. Petersen-Jones

**Affiliations:** 1 Department of Small Animal Clinical Sciences, College of Veterinary Medicine, Michigan State University, East Lansing, Michigan, United States of America; 2 Genetics Program, Michigan State University, East Lansing, Michigan, United States of America; 3 Department of Animal and Food Sciences, University of Wisconsin-River Falls, River Falls, Wisconsin, United States of America; 4 Health Information Technology, Michigan State University, East Lansing, Michigan, United States of America; 5 Department of Microbiology and Molecular Genetics, Michigan State University, East Lansing, Michigan, United States of America; Radboud University Nijmegen Medical Centre, The Netherlands

## Abstract

Retinal dystrophies in dogs are invaluable models of human disease. Progressive retinal atrophy (PRA) is the canine equivalent of retinitis pigmentosa (RP). Similar to RP, PRA is a genetically heterogenous condition. We investigated PRA in the Papillon breed of dog using homozygosity mapping and haplotype construction of single nucleotide polymorphisms within a small family group to identify potential positional candidate genes. Based on the phenotypic similarities between the PRA-affected Papillons, mouse models and human patients, *CNGB1* was selected as the most promising positional candidate gene. *CNGB1* was sequenced and a complex mutation consisting of the combination of a one basepair deletion and a 6 basepair insertion was identified in exon 26 (c.2387delA;2389_2390insAGCTAC) leading to a frameshift and premature stop codon. Immunohistochemistry (IHC) of pre-degenerate retinal sections from a young affected dog showed absence of labeling using a C-terminal CNGB1 antibody. Whereas an antibody directed against the N-terminus of the protein, which also recognizes the glutamic acid rich proteins arising from alternative splicing of the CNGB1 transcript (upstream of the premature stop codon), labeled rod outer segments. CNGB1 combines with CNGA1 to form the rod cyclic nucleotide gated channel and previous studies have shown the requirement of CNGB1 for normal targeting of CNGA1 to the rod outer segment. In keeping with these previous observations, IHC showed a lack of detectable CNGA1 protein in the rod outer segments of the affected dog. A population study did not identify the *CNGB1* mutation in PRA-affected dogs in other breeds and documented that the *CNGB1* mutation accounts for ∼70% of cases of Papillon PRA in our PRA-affected canine DNA bank. *CNGB1* mutations are one cause of autosomal recessive RP making the *CNGB1* mutant dog a valuable large animal model of the condition.

## Introduction

Retinitis pigmentosa (RP) is the leading cause of inherited blindness in humans affecting about 1 in 4,000 people [1]. It can be inherited in a dominant, recessive or X-linked fashion and shows considerable locus heterogeneity, with mutations in over 40 genes identified as causing non-syndromic RP (RetNet: https://sph.uth.edu/retnet/sum-dis.htm). Proteins encoded by these genes are necessary for a variety of functions within photoreceptors and their supporting cells. The age at onset and rate of progression of RP vary such that some patients have a history of night blindness from childhood while others may not notice symptoms until they are adults. The variability depends on the gene involved and the effect of the mutation on gene function, but there is also variability between patients with the same mutation [2], [3]. Rod photoreceptors are affected initially, resulting in loss of night (rod-mediated) vision and constriction of the visual fields. Loss of cone-mediated (daytime and color) vision may occur secondarily to rod-loss, even when RP is caused by a mutation of a gene exclusively expressed in rods, and can lead to complete blindness.

Retinal dystrophies analogous to RP occur in dogs, with reports of such conditions in over 100 different breeds [4]. The canine RP equivalent is known as progressive retinal atrophy (PRA) [5], [6]. The gene mutations underlying several forms of PRA have been identified and many have proven to be in genes analogous to those known to cause RP [7], [8], [9], [10], [11], [12], [13] or in some instances have suggested new candidate genes for investigation in RP patients [14], [15], [16].

Spontaneously occurring retinal dystrophies in canine models are of particular interest because the canine eye is similar in size to the human eye. This morphological similarity allows for identical surgical approaches for intravitreal and subretinal injection of therapeutic agents and testing for approaches such as implantation of intravitreal sustained-release devices. An additional advantage of canine models over rodent models is that the canine eye has regions of higher photoreceptor density (of both rods and cones), namely the area centralis and the visual streak that are somewhat analogous to the human macula [17]. In contrast, the retina of laboratory rodents lacks an equivalent region having an even density of photoreceptors across the retina [18]. Dogs with spontaneous mutations resulting in retinal dystrophies have proven to be important in preclinical assessment of therapies destined for use in human patients. For example, dogs with a mutation in *RPE65* as a model for Leber congenital amaurosis type II were crucial for preclinical proof-of-concept gene therapy trials [19] which led to phase 1/2 human clinical trials [20], [21], [22]. The RPE65 mutant dog and other dog retinal dystrophy models have subsequently been used in several other preclinical trials for retinal gene and drug therapy [19], [23], [24], [25], [26], [27], [28], [29], [30], [31]. Identification of the gene mutations underlying other forms of canine PRA may provide additional spontaneous canine models to allow study of disease mechanisms and proof-of-concept therapy trials.

The Papillon breed of dog was initially reported to have PRA in 1995 [32]. Studies of the phenotype of affected dogs suggested loss of rod electrophysiological responses but maintenance of cone-driven responses at least until late in the disease process [33], [34]. Our unpublished studies of PRA in Papillons show a wide range in age of onset. This phenotypic variability could either suggest within-breed locus heterogeneity or could merely be the result of background genetic or environmental influences.

In this study we report a frameshift mutation in *CNGB1* that is the cause of one form of PRA in Papillon dogs providing a large-animal model of autosomal recessive RP (RP45) due to *CNGB1* mutations.

## Results

### Phenotypic Description

**Figure 1:**
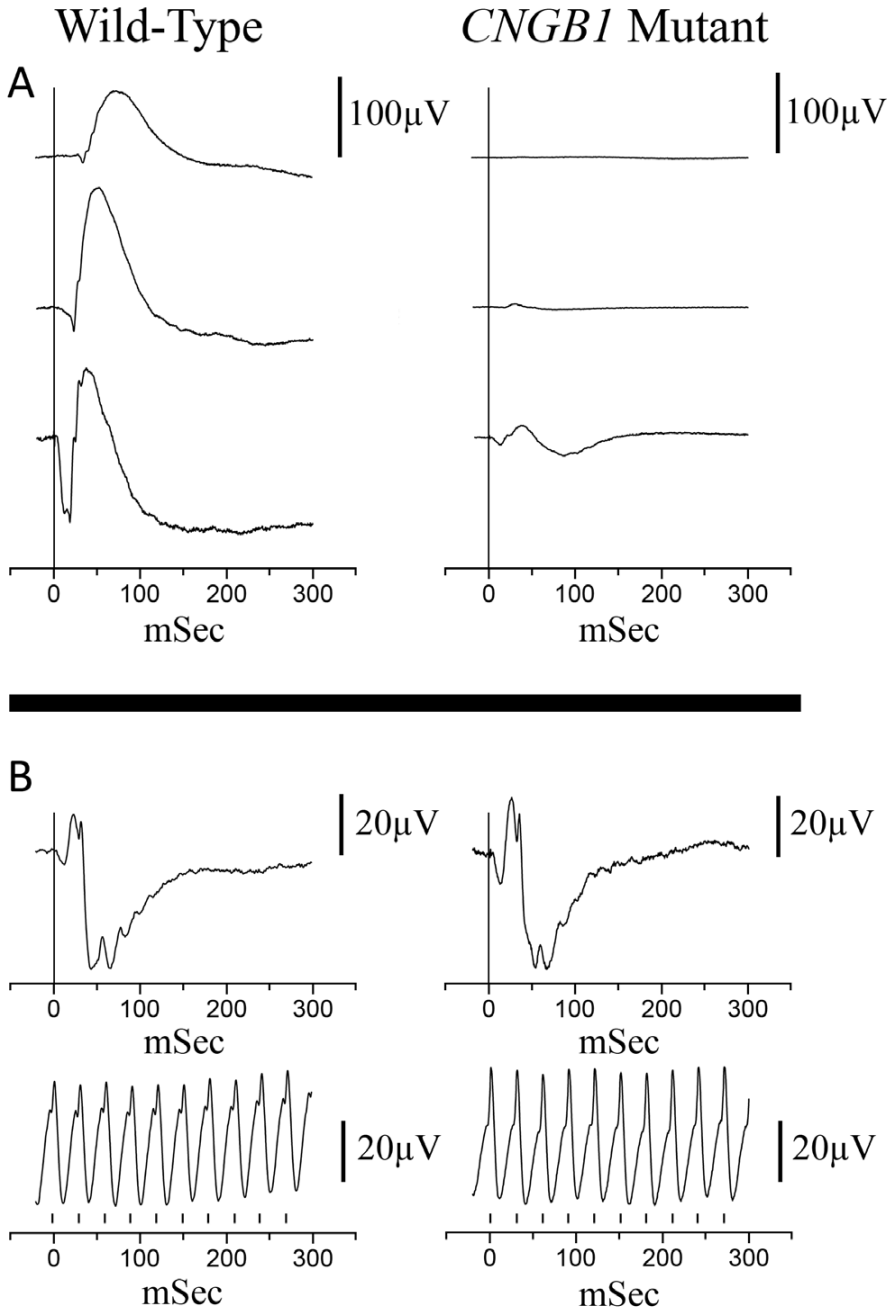
Representative ERG tracings from a normal control Papillon and a PRA-affected Papillon, both 10 weeks of age. A. Dark-adapted ERG recordings at −2.4, −1.2 and 0.4 log cdS/m^2^. B. Light-adapted flash and flicker (33 Hz) ERG tracings. Background white light of 30 cd/m^2^ and flash intensity of 0.4 log cdS/m^2^. The vertical bars on the flicker ERG indicates the flash timing.

**Figure 2:**
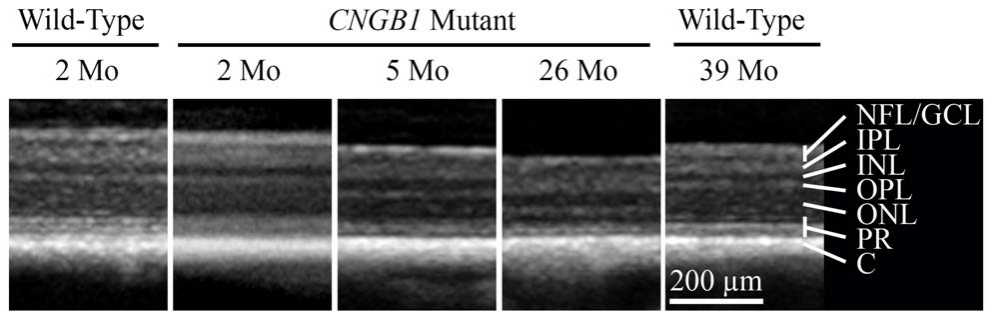
SD-OCT cross sectional images of the central retina from *CNGB1* mutant Papillons and wild-type controls. Note the progressive thinning of the outer nuclear layer of the retina in the affected animals. NFL/GCL – nerve fiber layer/ganglion cell layer; IPL – inner plexiform layer; INL – inner nuclear layer; OPL – outer plexiform layer; ONL – outer nuclear layer; PR – photoreceptor inner and outer segments; C – choroid. (Note the retinal pigment epithelium is not labeled).

DNA samples were collected from 23 PRA-affected Papillons and 119 unaffected Papillons. The dogs had all been examined by a veterinary ophthalmologist. For the affected dogs an ophthalmoscopic diagnosis was made between 10 months and 13 years of age (data not shown). A small breeding colony of PRA-affected Papillons was established, consisting of an affected female and two affected offspring. Electroretinography (ERG) showed markedly reduced or absent rod-mediated ERG responses from an early age with preservation of cone photoreceptor responses (Figure 1). Spectral Domain-Optical Coherence Tomography (SD-OCT) performed on PRA-affected Papillons (confirmed to have the *CNGB1* mutation described in this paper) showed that at the time the canine retina reaches maturity (approximately 8 weeks of age [35]) retinal layer thicknesses were comparable to a normal control (Figure 2A and 2B) and that affected dogs have a progressive thinning of the outer nuclear layer with age (Figure 2C & D).

### Mapping of the Papillon PRA Locus

**Figure 3:**
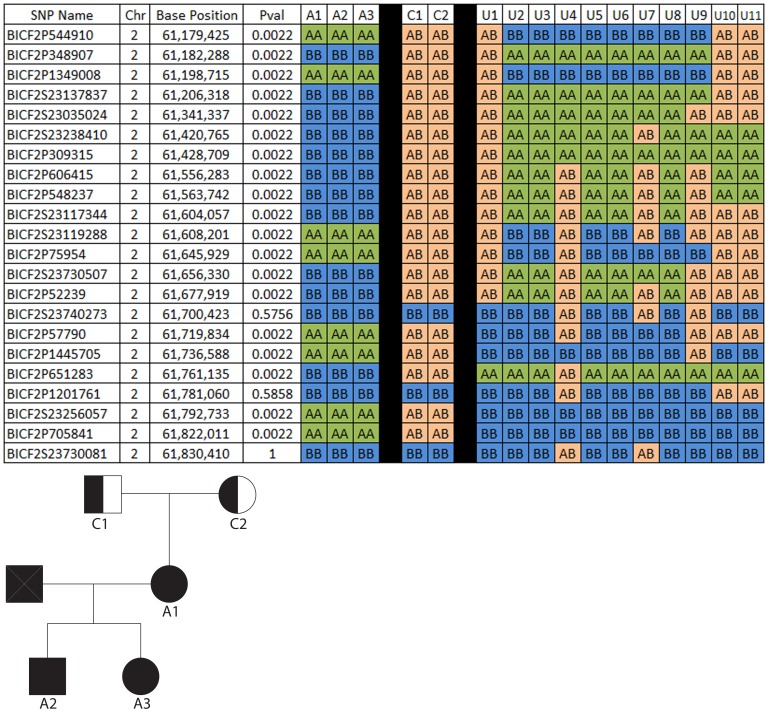
SNP analysis in the *CNGB1* region using a small family group of affected and unaffected Papillons. The SNPs are located in a 1.84 Mb region of homozygosity among the affected dogs (Only SNPs near the center of the region are shown). *CNGB1* is located at chr2:61,454,476–61,520,336 in the reference genome (canFam2.0). SNP genotypes are given in columns for each dog, with the family group data displayed to the left of the black back and all others to the right of the black bar. All affected dogs (1–3) share the same haplotype, obligate carriers (C1–2) each have one copy of the haplotype seen in the affected dogs and none of the unaffected dogs (U1–11) possess the haplotype seen in the affected dogs. A-Major allele, B-minor allele, as designated by Illumina (Illumin Inc, San Diego, CA).

An initial genome-wide association analysis performed using PLINK software [36] and including the genotypes from 23 Papillons (9 cases, 4 obligate carriers and 10 controls) yielded no significant associations (data not shown). Because within breed locus heterogeneity for PRA in dogs is a common occurrence, and because we and others had noted a wide range of age of onset [32] between the affected dogs, we suspected that more than one form of PRA may be segregating in the Papillon breed. Therefore, we analyzed the genotyping data from a small family group of 3 affected dogs within our breeding colony that we felt were very likely to share the same gene mutation (pedigree in Figure 3). We compared the genotyping results of the 3 affected dogs with that of 2 obligate carriers from the family and 11 control dogs (an additional control was included in this analysis). Our pedigree analysis supported an autosomal recessive mode of inheritance (data not shown) so we performed homozygosity mapping using a custom written computer program. The program was set to identify regions of homozygosity containing runs of at least six SNPs in the cases and for which the control animals did not share homozygosity (see Materials and Methods). This revealed 13 such regions of homozygosity greater than 1.5 Mb but only 4 of these regions contained obvious positional autosomal recessive RP candidate genes; *CNGB1* (CFA2), *RBP3* and *RGR* (CFA4), *RD3* and *CRB1*(CFA7) and *TULP1* (CFA12) (Table 1). Figure 3 shows a section of the run of homozygosity surrounding *CNGB1* and the *p*-values for each marker, resulting from a chi-square association test corrected for multiple testing (the full region of homozygosity is shown in Table S4). These four regions were then subjected to haplotype construction, and haplotypes were examined within the small family group (data not shown). Only in the CFA2 region did the affected dogs from this family have a unique extended haplotype which was not present in the homozygous state in control (non-obligate carrier) dogs. Obligate carrier dogs each possessed one copy of this haplotype. Furthermore, after comparing the phenotype of the PRA-affected dogs in our colony with that reported for human families and mouse models with *CNGB1* mutations, *CNGB1* was considered the strongest candidate. Based on the haplotype analysis and phenotypic information, *CNGB1* was selected to screen first for mutations.

**Table 1:** Regions of homozygosity above 1.5 MB from small Papillon family.

Chr	Start Position^1^	End Position^1^	Size of Region (bp)	arRP Candidate Genes^2^	Location of Candidate Genes^1^
6	3,330,209	12,765,968	9,435,759	–	–
28	10,867,526	19,744,585	8,877,059	–	–
4	34,171,819	42,900,776	8,728,957	*RGR*	ch4∶37,501,028–37,504,555
4	34,171,819	42,900,776	8,728,957	*RBP3*	ch4:38,165,164–38,175,385
21	19,922,213	28,084,112	8,161,899	–	–
7	7,812,042	14,771,908	6,959,866	*CRB1*	chr7:8,233,979–8,375,413
7	7,812,042	14,771,908	6,959,866	*RD3*	chr7:12,835,467–12,835,739
2	37,571,313	43,948,453	6,377,140	–	–
12	3,051,458	8,579,562	5,528,104	*TULP1*	chr12:7,639,870–7,647,308
7	3,000,316	7,077,039	4,076,723	–	–
37	23,673,475	26,349,135	2,675,660	–	–
14	8,954,008	11,562,422	2,608,414	–	–
32	11,212,687	13,539,152	2,326,465	–	–
2	60,980,617	62,826,928	1,846,311	*CNGB1*	chr2:61,454,476–61,520,336
14	37,878,684	39,484,021	1,605,337	–	–

1. Locations are all in respect to UCSC Genome Browser CanFam2.0 (http://genome.ucsc.edu).

2. arRP - autosomal recessive retinitis pigmentosa. Gene abbreviations: *RBP3* - retinol binding protein 3, *RGR* - retinal G protein coupled receptor, *RD3* - retinal degeneration protein 3, *CRB1* - crumbs homolog 1, *TULP1* - tubby like protein 1, *CNGB1* - cyclic nucleotide gated channel beta 1.

### Genomic Structure of Canine *CNGB1*

To establish the genomic structure of canine *CNGB1* we sequenced cDNA from a control canine retinal library and genomic DNA from a control Papillon and compared these to the published canine genomic sequence for this region (CanFam2.0). From these comparisons we deduced the intron/exon boundaries for *CNGB1* (Table 2) which differed from the predicted structure seen on the University of California, Santa Cruz (UCSC) Genome Browser [37] (http://genome.ucsc.edu/).

**Table 2:** Intron and exon boundries for canine CNGB1 gene.

	Location on Chr2^1^	Donor^2^	Intron (size)	Acceptor^2^	
Exon 1	61,454,476–61,454,528	ATCTGAGCAAgtaagtcagg	1 (2533 bp)	tgtcctacagGTGTCGGGAT	Exon 2
Exon 2	61,457,062–61,457,243	GGACTCCCTGgtaagagaat	2 (2069 bp)	aaccttccagCCACCTGAAG	Exon 3
Exon 3	61,459,313–61,459,364	GGCCCTCAGGgtgagtgccg	3 (288 bp)	tcttttccagAAATCCAGGA	Exon 4
Exon 4	61,459,653–61,459,725	AAGTGAACAGgtaccacacc	4 (772 bp)	tgtccctcagCAGTTCCAAC	Exon 5
Exon 5	61,460,498–61,460,618	CCCTGCTCAGgtacttctga	5 (89 bp)	gtcctggcagGCAGGAGCAC	Exon 6
Exon 6	61,460,708–61,460,750	GGGAGCTCAGgtgaggccag	6 (184 bp)	ttcttctcagATGGACTTGG	Exon 7
Exon 7	61,460,935–61,460,965	GGGACACCGGgtgagtcctc	7 (1283 bp)	ccctgtgcagGTCTGGGCCC	Exon 8
Exon 8	61,462,249–61,462,324	AACCTCCAAGgtaagtcaaa	8 (352 bp)	tctgatctagGACCAGAGAG	Exon 9
Exon 9	61,462,677–61,462,716	TTGGACACAGgtacagggag	9 (365 bp)	tgctgcttagAGCCCCCTGG	Exon 10
Exon 10	61,463,082–61,463,241	ACCCTGCCAGgtgagccccc	10 (852 bp)	ccacttgcagGTTGATGGCT	Exon 11
Exon 11	61,464,094–61,464,169	CAGGGAGCAGgtctgttctg	11 (1686 bp)	tcccatgcagGAGCCTGACT	Exon 12
Exon 12	61,465,856–61,465,892	GTGCAGACCAgtaagtgcct	12 (6320 bp)	tcccctgcagTCTGCATCCT	Exon 13
Exon 13	61,472,213–61,472,375	AGATGCCCAGgtgggagcca	13 (1405 bp)	ctcaactcagGAAGCTGCCC	Exon 14
Exon 14	61,473,781–61,473,861	AGGAGGCAAAgtaaggtgct	14 (5744 bp)	ggggtcacagTGTCCTGCTG	Exon 15
Exon 15	61,479,606–61,479,693	CTCCCAGCAGgtacggagcg	15 (685 bp)	ttgtctgcagGAGCTGCAGG	Exon 16
Exon 16	61,480,379–61,480,415	AGCCCAGAAGgtaggtgtgc	16 (5435 bp)	tctctccaagTGCCTGCTAC	Exon 17
Exon 17	61,485,851–61,486,010	GAACAGAAAGgtcacctttt	17 (2502 bp)	gtccttgcagGAAGAGGCTG	Exon 18
Exon 18	61,488,513–61,488,620	AAGGCACTGAgtgagtgggg	18 (2472 bp)	gtgtccacagTGGCCAGGAT	Exon 19
Exon 19	61,491,093–61,491,250	CCAAAGCCCTgtgagtccag	19 (1047 bp)	catcccacagCCCCGGCCAA	Exon 20
Exon 20	61,492,298–61,492,456	CCGCTGACCAgtgagtcctg	20 (883 bp)	ctccctgcagACCTGATGTA	Exon 21
Exon 21	61,493,340–61,493,548	AGACATCATTgtgagtcccg	21 (723 bp)	tttctttcagACAGACAAAA	Exon 22
Exon 22	61,494,272–61,494,322	TCGCTTTAAGgtgcgcgctg	22 (273 bp)	gggatttcagATGGACATGC	Exon 23
Exon 23	61,494,596–61,494,682	CTGTTTGAAGgtaggcttcc	23 (1879 bp)	ttcttcccagTACATGGCCT	Exon 24
Exon 24	61,496,562–61,496,626	ATGTTTACAGgtgagacaca	24 (808 bp)	tctcccgcagGGTCATCAGG	Exon 25
Exon 25	61,497,435–61,497,557	TGGGAAACAGgtgagccagt	25 (5025 bp)	ctctctctagTTACATTCGC	Exon 26
Exon 26	61,502,583–61,502,724	GATCGGACAGgtagctgggt	26 (704 bp)	ttgcccctagATGAGAGACG	Exon 27
Exon 27	61,503,429–61,503,588	GGCATGCTGGgtaagatggg	27 (1872 bp)	tcctttccagACGAGTCAGA	Exon 28
Exon 28	61,505,461–61,505,558	TCTCTTCCAGgtatggcccc	28 (100 bp)	ttggggacagGGCTGTGACC	Exon 29
Exon 29	61,505,659–61,505,742	GTGCAAGAAGgtgagtggcc	29 (3003 bp)	tctgtttcagGGGGAGATAG	Exon 30
Exon 30	61,508,746–61,508,864	GAGAAATAAGgtcagagggg	30 (258 bp)	tctaccccagCTTACTGGCT	Exon 31
Exon 31	61,509,123–61,509,269	AGAAGGCCAGgtacattttt	31 (6620 bp)	tttctttcagGCGCATGCTG	Exon 32
Exon 32	61,515,890–61,516,109	GCTGGAACAGgtaagatggt	32 (2609 bp)	tggattttagGCCAAGAGCT	Exon 33
Exon 33	61,518,719–61,519,006	GGCCGAGTGA - 3′UTR			

1. Locations are all in respect to UCSC Genome Browser CanFam2.0 (http://genome.ucsc.edu).

2. Capital letters are exonic DNA sequences and lower case bases are intronic regions.

End of coding region marked in Exon 33 row by underlined TGA.

### Sequence Analysis of *CNGB1*

The full coding region of canine *CNGB1* was sequenced from the cDNA of a control canine retinal library (cDNA sequence submitted to GenBank KC527595). Sequencing cDNA from the control dog revealed three single nucleotide variants (SNVs), at locations c.3378C>A, c.3440T>C and c.3534G>A, and one previously reported single nucleotide polymorphism (SNP) at location c.151G>A (rs22870569). *CNGB1* exons and exon/intron boundaries were sequenced from genomic DNA from control and affected Papillons. This revealed the same SNVs and SNP as seen in the sequenced cDNA but also contained two additional variants; a previously described SNP (c.27G>A, rs22870567) and a SNV allele that segregates with the affected phenotype (c.215C>T; p.P72L). PolyPhen-2 predicts that this is a benign change in amino acid (HumVar 0.412) [38].

**Figure 4:**
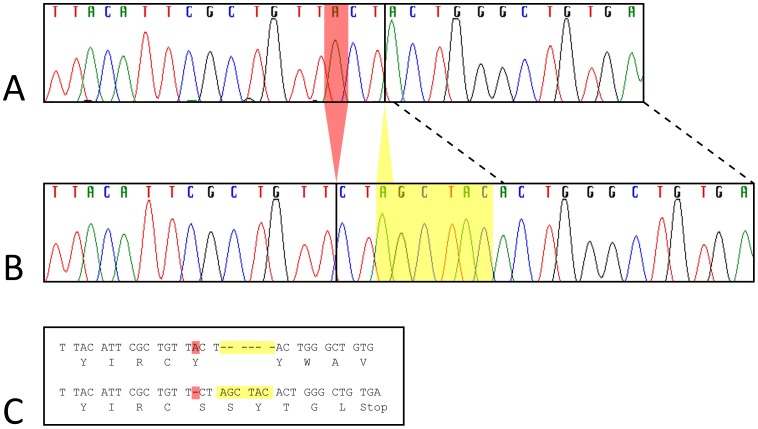
Papillon mutation in *CNGB1* exon 26. Sanger dideoxy-sequencing traces for part of *CNGB1* exon 26 are shown for an unaffected (A) and an affected (B) Papillon. Panel C shows the codon and amino acid alignment inferred from the traces in panels A and B, for the unaffected sequence (top) and affected Papillon mutation sequence (bottom). The complex Papillon mutation includes a 6 bp AGCTAC insertion between reference bases chr2:61,502,599–61,502,600 (yellow highlight in panel C and within yellow box in panel B) and an adenine deletion at chr2: 61,502,597 (red highlight in panel C and red triangle in panel B). The deletion causes a frameshift and premature stop codon within seven residues, including the two new, inserted codons.

The affected Papillon had a frameshift mutation in exon 26. This consisted of a 1 bp deletion (chr2: 61,502,597; c.2387delA) and a 6 bp insertion (between chr2:61,502,599–61,502,600; c. 2389_2390insAGCTAC). This mutation (c.2387delA;2389_2390insAGCTAC, which for simplicity we will refer to as *CNGB1-fs26*) is predicted to result in a premature stop codon, 17 bp downstream and is present in affected Papillons but not in unaffected Papillons or in the canine reference genome (UCSC Genome Browser CanFam2.0) (Figure 4).

Table 3 shows the SNVs and SNPs detected and Figure S1 shows the predicted canine protein amino acid sequence and alignment with other species. Primers for sequencing canine gDNA and cDNA are supplied in Table S1 and Table S2, respectively.

**Table 3:** Single nucleotide variant (SNV) locations in genomic Papillon CNGB1.

Location	Position on Chr2^1^	cDNA change	Reference allele^2^	Variant allele^3^	cDNA bp	Protein change^4^	SNP number^5^
Exon 2	61,457,096	c.27G>A	G	A	G	–	rs22870567
Exon 2	61,457,220	c.151G>A	G	A	A	p.E69K	rs22870569
Exon 3	61,459,353	c.215C>T	C	T	C	p.P72L	–
Exon 33	61,518,754	c.3378C>A	C	A	A	–	–
Exon 33	61,518,816	c.3440T>C	T	C	C	p.L1165P	–
Exon 33	61,518,910	c.3534G>A	G	A	A	–	–

1. Locations are all in respect to UCSC Genome Browser CanFam2.0 (http://genome.ucsc.edu).

2. Reference allele from UCSC Genome Browser CanFam2.0.

3. Variant allele from sequenced gDNA of Papillons.

4. Protein change due to the SNV found in either Papillons and/or cDNA. Change is in respect to CanFam2.0.

5. SNP number from Broad Institute SNP collection (http://www.broadinstitute.org/mammals/dog/snp2).

A genotyping assay for the mutation was developed (methods S1). The assay was used to genotype 20 Papillons that had been diagnosed by a veterinary ophthalmologist to have PRA (not including purpose-bred colony dogs) and 119 Papillons whose owners reported no abnormal vision (Table 4). The mutation was identified in the homozygous state in 13 of 20 Papillons that had been diagnosed with PRA. Of the phenotypically normal Papillons, none were homozygous for the mutation and 20 were heterozygous for it. This indicates a 16.5% carrier rate, and a mutated allele frequency of ∼31% in the Papillon breed, however, ascertainment bias almost certainly falsely inflates these values. In addition, we genotyped 33 dogs from 8 different breeds that had been diagnosed with PRA and 66 dogs from 9 different breeds that were clinically normal, none of the dogs of non-Papillon breeds had the mutation (Table S3).

**Table 4:** PRA type 1 genotypes and clinical status for 139 Papillons.

Genotype^1^	Clinical Status		
	PRA affected^2^	Unaffected	Total
CNGB1 M/M	13	0	13
CNGB1 M/+	3	20	23
CNGB1+/+	4	99	103
Total	20	119	139

1. Genotyping results: (+/+) means wild-type *CNGB1* sequence. M = mutant (c.2387delA;2389_2390insAGCTAC) genotype.

2. Not including colony dogs to avoid inflation of mutation presence in the general population of Papillon dogs.

### Immunohistochemistry Shows Lack of Detectable Full-length CNGB1 Protein in Affected Retina

To confirm that the *CNGB1-fs26* mutation does disrupt CNGB1 expression in the homozygous animal, we performed immunohistochemistry (IHC) on retinal sections from an 8 week-old PRA-affected Papillon from the breeding colony that was homozygous for the *CNGB1-fs26* mutation and compared it to retinal sections from an 8 week-old normal dog that was confirmed not to have the *CNGB1-fs26* mutation.

**Figure 5:**
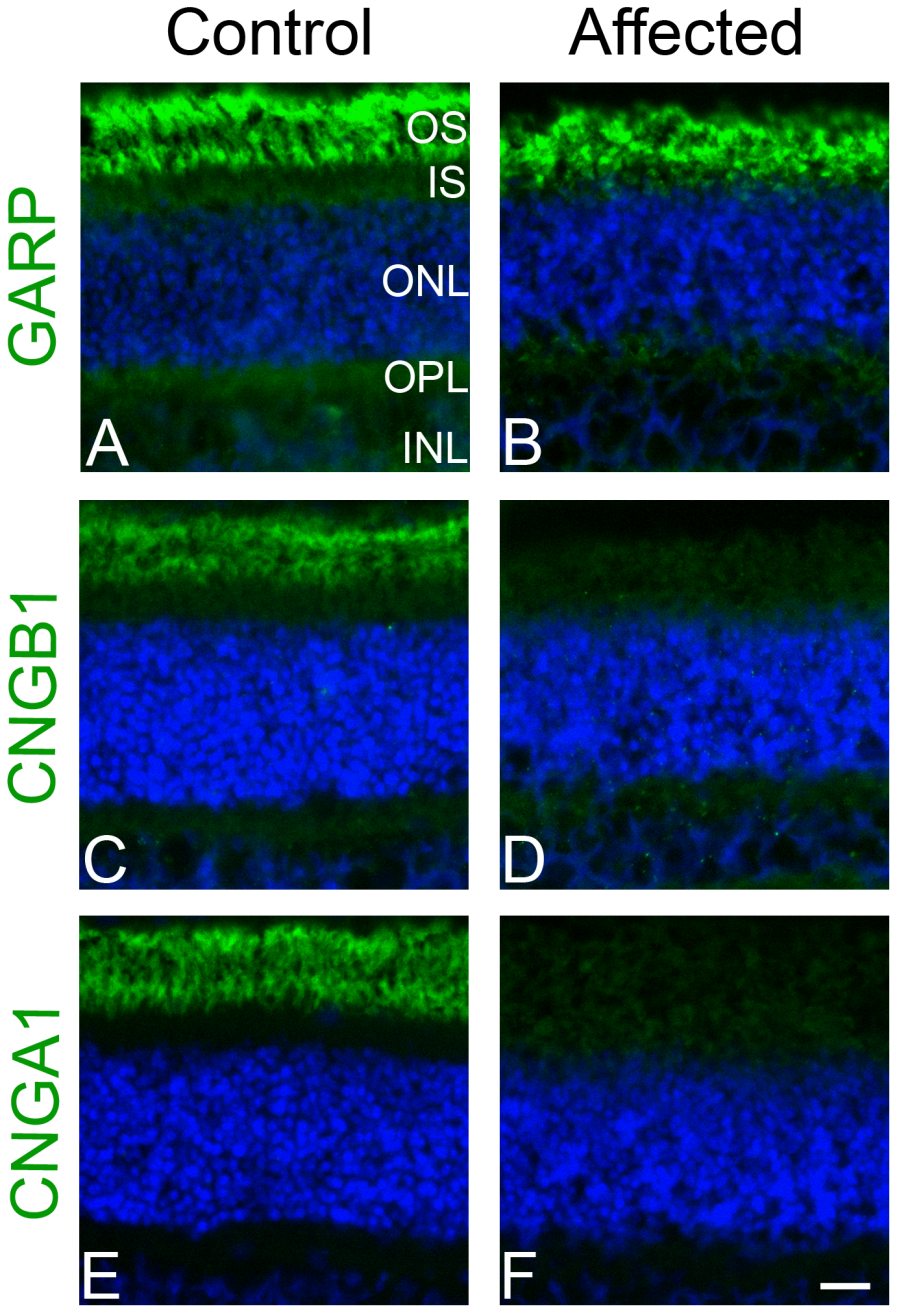
Immunohistochemistry on frozen retinal sections from age (8 wk) and sex matched (female) control and affected dogs. Images on the left are from the control dog and images on the right are from the affected dog. Panels A and B are stained with GARP-CNGB1 N-terminal antibody (green) and DAPI (blue). GARP proteins are present in both the control and affected samples. Panels C and D were stained with CNGB1 C-terminal antibody (green) and DAPI. CNGB1 full length protein is not detected in the affected sample. Panels E and F are stained with CNGA1 antibody (green) and DAPI. CNGA1 is not detected in the affected sample, presumably due to necessity for CNGB1 to form viable channels and normal trafficking. Size bar: 20 µm. OS- photoreceptor outer segment, IS – photoreceptor inner segment, ONL – outer nuclear layer, IPL – inner plexiform layer, INL – inner nuclear layer.

*CNGB1* in other species codes for multiple transcripts via alternative splicing [39], [40]. The *CNGB1* locus has been described to code for four sensory transcripts; three retinal transcripts and one olfactory sensory transcript, as well as other splice variants expressed in kidney, brain, testes and spermatozoa [41], [42], [43], [44]. The 5′ portion of the gene encodes two glutamic acid rich proteins (GARPs) while the full-length transcript encodes the CNGB1 protein. The position of the *CNGB1-fs26* mutation is predicted to allow normal expression of the two GARPs but to disrupt production of the full-length CNGB1 protein. To test this prediction we used two CNGB1 antibodies: one that targets the amino terminal (GARP region) of CNGB1 and the GARPs and a second antibody that targets the carboxyl end of CNGB1 downstream of both the GARP region and the predicted premature stop codon in the mutant canine *CNGB1* gene. The results from this study showed that while the rod outer segments of the wild-type retina were labeled by both antibodies (Figure 5 A, C), the rod outer segments of the PRA-affected Papillon were labeled with the amino terminal antibody but not the carboxyl terminal antibody (Figure 5 B, D). This provides strong evidence that the mutation disrupts production of full-length CNGB1 protein while still allowing expression of GARPs as predicted from the *CNGB1-fs26* mutation.

We also performed IHC with a CNGA1 antibody. The rod cGMP-gated channel consists of both CNGA1 and CNGB1 subunits. Studies in mouse models have shown that lack of CNGB1 also disrupts trafficking of CNGA1 to the outer segments resulting in very reduced or absent CNGA1 protein [45], [46]. As predicted by the mouse model, the retinal sections for the affected Papillon showed lack of detectable CNGA1 protein while in the retinal sections from the normal dog CNGA1 was appropriately expressed and correctly targeted to the rod outer segments (Figure 5 E, F).

## Discussion

We used homozygosity mapping of SNP microarray genotyping data from PRA-affected Papillons to identify regions where the affected dogs had runs of homozygosity spanning greater than 1.5 Mb and for which control dogs showed allelic variability. Positional candidate genes were identified mapping to those regions and haplotype analysis revealed only one region (on CFA2) in which obligate-carrier dogs had one copy of the haplotype observed in affected dogs, and none of the unrelated control dogs were homozygous for the haplotype observed in affected dogs. *CNGB1* was selected as the most promising positional candidate gene because of its location within the CFA2 region and of the similarity of the phenotype of *CNGB1* retinal dystrophy in mice and humans to that of the PRA-affected Papillons in our breeding colony (early lack of rod function and yet a slow retinal degeneration). The canine genome assembly (CanFam2.0) on the UCSC Genome Browser had incorrect exon predictions for *CNGB1*. We established the normal gene structure by sequencing cDNA from a retinal library developed from a control dog (GenBank KC527595). Sequencing of the confirmed exons and nearby flanking intronic regions of *CNGB1,* in affected and phenotypically normal Papillons, revealed a frameshift mutation *(CNGB1-fs26)* that segregated with disease status in our breeding colony and was not present in the homozygous state in any unaffected Papillons. A missense variation was also detected in the affected dogs (p.P72L). This converts a proline to leucine but at a residue that in some species is a leucine and in others a proline, making it unlikely that this would have a major effect on the function of CNGB1 in the dog [47]. Furthermore, this amino acid change was predicted to be benign by the PolyPhen-2 program [38].

Cyclic nucleotide gated (CNG) channels are necessary for normal phototransduction. There are different CNG channels encoded by paralogous genes specific to the rod and cone photoreceptors [41]. The rod CNG channel is a heterotetrameric protein consisting of three CNGA1 subunits and one CNGB1 subunit [48], [49], [50]. In dark conditions, the rod has higher levels of cyclic guanosine monophosphate (cGMP) which act to open a proportion of the CNG channels allowing an influx of cations, resulting in depolarization of the rod photoreceptor. Light stimulation triggers the phototransduction cascade activating the cGMP phosphodiesterase that hydrolyzes cGMP lowering its concentration and leading to closure of the CNG channels. This halts the influx of cations through the channel which, coupled with the continued action of other ion pumps in the cell membrane, results in the light-induced hyperpolarization of the rod photoreceptor.

CNGA1 can form functional channels *in vitro* without CNGB1 [51], [52], [53], while CNGB1 on its own does not form a functional channel. However, *in vivo* CNGB1 is important for rod CNG formation and normal functionality of the channel [45], [46]. The *CNGB1* gene codes for three splice variants in the retina that encode the soluble glutamic acid rich proteins 1 and 2 (GARP1 and GARP2) and a full length CNGB1 protein [39]. The full length transcript in the retina is known as *CNGB1a*. Full length CNGB1 protein in the retina consists of an N-terminal “GARP region” and a C-terminal “channel domain”. GARP1 is of low abundance whereas GARP2 is more highly expressed [46]. The functions of the GARP subunits are still being explored. GARP2 does bind to PDE6 and may act to reduce dark level noise [54]. It is also postulated to have a structural role in the rod outer segment [46] where it interacts with peripherin-2 at the rod outer segment disk rim [55]. As discussed further below GARP2 may also play a role in control of the opening of the CNG channel [56]. Other than these three splice variants expressed in the retina, *CNGB1* also has splice variants expressed in the olfactory epithelium (*CNGB1b* –which encodes a shorter protein than expressed in the retina lacking the GARP region), kidney, brain and testes [41], [42], [43], [44]. Two mouse gene targeting models, *CNGB1X-1* and *CNGB1X-26*, have been created to study the functions of these proteins in the retina.

The *CNGB1X-1* mouse knockout model, which lacks all retinal *CNGB1* products, has shown that GARPs are necessary in outer segment disk development and the structural integrity of the rod outer segments [46]. Interestingly, the *CNGB1X-1* mice have a weak rod response that is detectable on single cell recording. This activity originates from a low level of homomeric CNGA1 channel formation in the rod outer segments. Despite the presence of the channels and the resulting weak light-induced rod response, these mice have severe rod photoreceptor degeneration. It was suggested that degeneration may be due to rod structural, rather than functional, failure [46]. Another mouse model, *CNGB1X-26,* has an engineered mutation that leads to a deletion of exon 26 resulting in a frameshift that introduces a premature stop codon in the first triplet in exon 27 of *CNGB1.* The *CNGB1X-26* mouse has a complete absence of mRNA for full-length CNGB1 and thus a lack of the CNGB1 protein but still produces GARP proteins [45]. These mice have normal development of the rod outer segments and a slower retinal degeneration than the *CNGB1X-1* mouse.

The premature stop codon in the *CNGB1X-26* mouse is positioned at a site homologous to 10 codons upstream of the premature stop codon predicted in the *CNGB1-fs26* mutation identified in this study. While we anticipate that the premature stop codon in *CNGB1* in the PRA-affected Papillon will lead to nonsense-mediated decay of the abnormal *CNGB1* mRNA and a complete absence of the CNGB1 protein, as was reported for the *CNGB1X-26* mouse [45], rather than production of a truncated protein, further studies are required to confirm this.

IHC using an antibody that binds to the carboxyl end of CNGB1 confirmed the lack of full-length CNGB1 in the rod outer segments of a young affected Papillon prior to photoreceptor degeneration. The *CNGB1-fs26* mutation is not predicted to affect expression of the GARPs, and IHC using an antibody that recognizes GARPs as well as the GARP region of the full-length CNGB1 labeled the rod outer segments of the affected Papillon suggesting that GARPs and/or a truncated CNGB1 product are expressed. If the truncated mRNA avoids degradation and allows the production of a truncated protein, it would be missing domains essential for channel gating and cyclic nucleotide binding and would therefore not be expected to function normally. IHC also showed a lack of detectable CNGA1 protein in the rod outer segments in the young affected Papillon. Similar IHC results were observed in the *CNGB1X-26* mouse. It was suggested that the GARPs may have a dominant-negative effect on the transport of CNGA1 to the outer segment accounting for the lack of homomeric CNGA1 channels in the outer segments of *CNGB1X-26* mice (which have GARPs) contrasted with the presence of low levels of CNGA1 in outer segments of *CNGB1X-1* mice (which lack GARPs) [46]. The reduced level of CNGA1 in the outer segment of the *CNGB1X-1* mouse is probably a reflection of the importance of CNGB1 in transport of CNGA1 to the outer segment [46]. A region in the N-terminal section of the CNGB1 subunit (bovine amino acids #677-764) has been described to interact and promote protein-protein interactions with the CNGA1 subunit C-terminal region; this region is upstream of the *CNGB1-fs26* mutation and is not predicted to be effected by this mutation [57], [58]. As would be expected in animals with a lack of rod CNG channels, the ERG changes in the affected Papillons indicate a lack of rod function. Specifically, prior to a thinning of the outer nuclear layer, there was an elevated dark-adapted ERG threshold with only a recordable response to light intensities above cone response threshold, a marked reduction in a- and b-wave amplitudes for the dark-adapted brighter flashes that elicit a mixed rod cone response in normal dogs.

The *CNGB1X-26* mutation also lacks expression of the *CNGB1* splice variant expressed in the olfactory epithelium (*CNGB1b*). These mice show absence of proper channel localization and delayed olfaction [59]. More detailed phenotypic characterization of the dog model is required to ascertain how closely the canine phenotype, both retinal and olfactory, mimics the *CNGB1X-26* mouse model.

Mutations in *CNGB1* have been identified in human patients with autosomal recessive RP (RP45) and are reported to account for ∼4% RP of cases [1]. In 2001, Bariel et al. reported a consanguineous French family with RP that they mapped to an interval containing *CNGB1* [60]. They then identified a missense mutation that converted an evolutionarily conserved glycine to valine (c.2978G>T; p.G993V) which they predicted would alter the cyclic nucleotide-binding domain (CNBD). The effect of this mutation was further elucidated in an elegant study reported by Michalakis et al [56]; they showed that the p.G993V mutation prevented binding of cGMP and that binding is required for removal of an inhibitory effect that the GARP domain and also GARP2 has on channel opening, such that if cGMP cannot bind to CNGB1 the CNG channel is silent [56]. More recently resequencing of candidate RP genes led to the identification of a simplex RP patient homozygous for a missense substitution in *CNGB1* (c.2957A>T; p.N986I) resulting in substitution of a conserved amino acid 7 codons upstream of the mutation identified by Bariel et al. and also in the CNBD [61]. Kondo et al. used homozygosity mapping to screen known arRP genes in Japanese RP patients and in one patient identified a mutation at the donor site of exon 32 (c.3444+1G>A) of *CNGB1* [62]. Subsequently, Becirovic et al. performed exon trapping experiments to investigate the effect of the mutation. Their studies suggest that the mutation leads to skipping of exon 32 and replacement of the last 170 amino acids by 68 unrelated amino acids [63]. The probands in the Bariel et al. and Kondo et al. studies had night blindness from a young age and were diagnosed with RP in their 30′s [60], [62].

The *CNGB1-fs26* mutation identified in Papillon dogs was present in 13 of the 20 PRA-affected Papillons tested. This suggests that there is at least one additional PRA locus segregating within the breed. Within-dog breeds genetic heterogeneity for PRA is becoming more evident [13], [16]. Additional studies will be required to find the gene mutation(s) responsible for the other form(s) of PRA segregating in Papillons.

The early onset of loss of rod function in the *CNGB1-fs26* mutant dog, coupled with a slow retinal degeneration that we have observed in our colony dogs, seems to accurately parallel the described disease course in human patients as well as the comparable mouse model (*CNGB1X-26*). Recently recombinant adeno-associated viral vector-mediated gene therapy to deliver a normal copy of *CNGB1* to *CNGB1−/−* mice was reported to allow for CNG channel formation, restoration of rod function and retinal morphological preservation [64]. The early loss of rod function and yet slow rod photoreceptor loss in the animal models suggests that *CNGB1* RP is a good target for gene augmentation therapy. The *CNGB1*-*fs26* mutant dog promises to be a valuable model for preclinical trials of such therapy.

## Materials and Methods

### Ethics Statement

All procedures were in compliance with the ARVO statement for the Use of Animals in Ophthalmic and Vision Research and approved by the Michigan State University Institutional Animal Care and Use Committee (AUF number 05-11-106-00; Institutional NIH/PHS Animal Welfare Assurance number A3955-01).

### Electroretinography

To assess rod and cone photoreceptor function, electroretinograms (ERGs) were recorded using a modification of a previously described technique [26]. Briefly, ERGs were recorded using an Espion E2 Electrophysiology system with ColorDome Ganzfeld (Diagnosys LLC, Lowell, MA) and bandpass set between 0.5 and 500 Hz. Dogs were dark-adapted for one hour and anesthetized with injectable propofol (10 mg/kg PropoFlo, Abbott Animal Health, North Chicago, IL), intubated and maintained on inhaled 1 to 2% isoflurane (Isoflo, Abbott Laboratories, North Chicago, IL) delivered in oxygen. The ERG assessment consisted of three dark-adapted flash intensities at −2.4, −1.2 and 0.4 log cdS/m^2^ to record rod and mixed rod-cone responses. This was followed by light adaptation at 30 cd/m^2^ for 10 minutes and recording of a light-adapted response to a 0.4 log cdS/m^2^ flash and then 33 Hz flicker responses to the same intensity both superimposed on the same background light.

### Spectral Domain-Optical Coherence Tomography

Assessment of retinal morphology was performed by Spectral Domain-Optical Coherence Tomography (SD-OCT; Spectralis OCT+HRA Heidelberg Engineering Inc., Heidelberg, Germany). Dogs were anesthetized as described for ERG, the pupil dilated with 1% topical tropicamide (Mydriacyl, Alcon Laboratories, Honolulu, HI, USA), a lid speculum fitted and the eye positioned in primary gaze using a stay suture in the inferior perilimbal conjunctiva. High-resolution cross-section images obtained by line and volume scanning and images from the same region of the central retina of affected and control (wild-type) Papillons were assessed.

### Animal Use and Sample Collection

A pregnant female Papillon dog that had been diagnosed with PRA and had been mated with a PRA-affected Papillon stud dog was donated to the Michigan State University Comparative Ophthalmology laboratory with the consent of the owner to allow the study of the phenotype of PRA in the breed. This female and her offspring were used to establish a small breeding colony of dogs. The colony was kept under standard laboratory housing with 12:12 hr light:dark cycles.

Blood samples from client-owned Papillon dogs were donated with owner consent. DNA was extracted from blood samples using a commercial DNA extraction kit with a modified protocol (Qiagen Sciences, Germantown, MD). Briefly, a red blood cell lysis buffer (0.32 M sucrose, 10 mM Tris, 5 mM MgCl_2_) was added in a 2 step fashion to whole blood (3X volume and then 2X volume, respectively). Cell lysis solution (Qiagen Sciences, Germantown, MD) was added to lyse the white blood cells followed by addition of protein precipitation solution (Qiagen Sciences, Germantown, MD), isopropanol DNA precipitation and a 70% ethanol wash step.

The retina from a mixed breed dog was dissected from an enucleated eye and placed in an RNA stabilization buffer (RNAlater, Qiagen Sciences, Germantown, MD) and stored in a −80°C freezer until RNA extraction. RNA was extracted using an RNEasy kit according to manufacturer’s protocol (Qiagen Sciences, Germantown, MD). cDNA was made from mRNA using a 3′ RACE kit according to manufacturer’s protocol (Invitrogen, Carlsbad, CA).

### Genome-Wide Association Mapping

Twenty-four Papillons (9 cases, 15 controls) were genotyped for 173,662 single nucleotide polymorphisms (SNPs) using Illumina Canine HD BeadChips. Initial genome-wide association analysis was conducted using the genome analysis toolset PLINK [36]. SNPs with a minor allele frequency (MAF) of <5% and with missing genotype calls of >10% were removed from the analysis. The final data set consisted of 116,235 markers. All 24 individuals genotyped successfully for over 90% of the SNPs and were retained in the analysis. One of the control dogs was removed from the PLINK analysis but was used in the run of homozygosity analysis. The final genotyping rate was >99.8%. Chi-square association mapping was conducted in PLINK, and correction for multiple testing was achieved using the Max(T) permutation procedure (10,000 permutations) in PLINK.

### Custom Sorting Program and Haplotype Construction

Homozygosity mapping was performed using a custom sorting program. An algorithm was written to search for blocks of SNPs where there was a difference in the calls between the affected and unaffected dogs. The high quality SNP data, generated from PLINK, was imported to a Microsoft SQL Server 2008R2 database (Microsoft Corporation, Redmond, WA) to take advantage of its efficient set theory based querying mechanism. The received data was formatted with dog identifiers as columns and SNPs as rows. Indexing the data provided a method to measure continuity. The query used scalar-valued functions to assess the criteria described below and attached a flag to each row identified. For each individual SNP, if the affected dogs shared the same homozygous genotype, then the unaffected dogs were compared and rows identified where 95% of the unaffected dogs did not share the same genotype as the affected dogs. Upon those criteria being met, the affected and unaffected groups were considered ‘different’ by the algorithm. Identified SNPs were then sorted into groups formed by having a level of adjacency of at most four SNPs apart. Only sections with 6 or more SNPs meeting the above criteria were marked for further analysis. These regions were then sorted by size of the region and all regions 1.5 MB or larger were inspected for arRP candidate genes using the University of California, Santa Cruz (UCSC) Genome Browser [37] (http://genome.ucsc.edu/). Four regions of particular interest (on CFA2, CFA4, CFA7, and CFA12) were then subjected to haplotype construction, using fastPHASE [65]. Haplotypes were manually examined for shared regions in related affected family members.

### DNA Sequencing

The UCSC Genome Browser (http://genome.ucsc.edu/) CanFam2.0 was used in conjunction with the cDNA sequences to identify the exons for the *CNGB1* gene. Primers (Table S1) were designed flanking the entire exon and the splice sites using Primer3 (http://frodo.wi.mit.edu/). Sanger dideoxy-sequencing was done by an ABI 3730 Genetic Analyzer (Applied Biosystems, Inc., Foster City, CA) at Michigan State University’s Research Technology Support Facility.

### CNGB1 Genotyping Assay

A restriction enzyme digest was designed to quickly screen dogs for the mutation in *CNGB1* (see methods S1). This assay was used to test for the presence of the mutation in 139 Papillon dogs, 33 PRA affected dogs from 8 different breeds and 66 unaffected dogs from 9 different breeds.

### Immunohistochemistry

A PRA-affected female Papillon from the research colony and an unaffected mixed breed female control dog were humanely euthanized at 8 weeks of age. The eyes were enucleated and the right eye was fixed in paraformaldehyde following a previously describe protocol [66].

Frozen sections were immunolabeled with either a rabbit anti-mouse CNGB1 N-terminal (kindly provided by Dr. Stephen Pittler), rabbit anti-human CNGB1 C-terminal (Sigma-Aldrich, St Louis, MO) or mouse monoclonal CNGA1 ([67] kindly provided by Dr. Bob Molday) antibody. Sections were blocked with 10% horse serum (Sigma-Aldrich, St Louis, MO) for 2 hours at room temperature and labeled with primary antibodies (dilutions of: N-terminal CNGB1 1:100, C-terminal CNGB1 1:300 and CNGA1 1:10) overnight at 4°C. Secondary antibodies (anti-rabbit or anti-mouse Alexa Fluor 488, 1∶500) (Invitrogen Molecular Probes, Carlsbad, CA) were placed on the sections for 2 hours at room temperature. All sections were counterstained with the nuclear stain DAPI (Invitrogen Molecular Probes, Carlsbad, CA).

Sections were imaged using a fluorescent microscope (Nikon Eclipse 80i, Nikon Instruments Inc., Melville NY) using commercial image capture software (MetaVue, Molecular Devices, Sunnyvale CA).

## Supporting Information

Figure S1 **CNGB1 amino acid alignments.** Sequence alignments performed using muscle alignment in SeaView software ([68]. Single nucleotide polymorphism and variants found in Papillon gDNA sequencing are numbered (1: p.E69K, 2: p.P72L, 3: p.L1165P) and the changed amino acid is underlined. 2: p.P72L is the SNV that has only been seen in Papillons with the mutation and is marked with red text. The *CNGB1X-26* mouse stop codon is highlighted in red. The Papillon mutation is highlighted in green. The human mutations are marked in yellow (highlighted N [61] G [60]and arrow which represents a splice mutation [62]). Epitopes for N-terminal antibody (mouse, highlighted in purple) and C-terminal antibody (human, highlighted in teal).
(DOCX)

Table S1 **Primers for genomic DNA sequencing.**
(XLS)

Table S2 **Primers for cDNA sequencing.**
(XLS)

Table S3 **Non-Papillon breeds tested for** ***CNGB1*** **mutation.**
(XLS)

Table S4 **The entire region of homozygosity surrounding the site of the** ***CNGB1*** **mutation.**
(XLS)

Methods S1 (DOCX)
