# Supplementary material for: A Large Animal Model for CNGB1 Autosomal Recessive Retinitis Pigmentosa
Source: PLoS One. 2013 Aug 19;8(8):e72229. doi: 10.1371/journal.pone.0072229 (PMC3747135; doi:10.1371/journal.pone.0072229)
Supplement: Figure S1 — CNGB1 amino acid alignments. Sequence alignments performed using muscle alignment in SeaView software ([68]. Single nucleotide polymorphism and variants found in Papillon gDNA sequencing are numbered (1: p.E69K, 2: p.P72L, 3: p.L1165P) and the changed amino acid is underlined. 2: p.P72L is the SNV that has only been seen in Papillons with the mutation and is marked with red text. The CNGB1X-26 mouse stop codon is highlighted in red. The Papillon mutation is highlighted in green. The human mutations are marked in yellow (highlighted N [61] G [60]and arrow which represents a splice mutation [62]). Epitopes for N-terminal antibody (mouse, highlighted in purple) and C-terminal antibody (human, highlighted in teal). (DOCX) [file pone.0072229.s001.docx]

**Supplemental Figure 1. CNGB1 amino acid alignments.**

canine MLSWVQRVLP QPPGTPQKTK VEEEGGADPE ---------- ---------- ---------- ----PEPEPE

human MLGWVQRVLP QPPGTPRKTK MQEEEEVEPE ---------- ---------- ---------- ----PEMEAE

mouse MLGWVQRVLP QPPGTPQKT- -VETAGPQPE TESKPEANPQ PEPEPQQEPE PEPEPEPEPE PEPEPEPEPE

rat MLGWVQRVLP QPPGTPQKT- -EEGAGPQPE TESKPEANPQ PEPEVQ---- ---------- --PEPEPEPE

bovine MLGWVQRVLP QPPGTPQKTK -QEEEGTEPE ---------- ---------- ---------- ----PELEPK

********** ****** ** * ** ** *

1 2

canine VKQEPELEPE TALEKAEQGD SLPPEEPL-- EEVAAADLGP QEIQEAALSP PTSLQAQITV APEVNSSSND

human VEPEPN--PE EAETESE--- SMPPEESFKE EEVAVADPSP QETKEAALTS TISLRAQGAE ISEMN-SPSR

mouse PEPEPEPVPE EAPPEVQ--- ALPPEEPMEG EGEAEAGPSL QETQVADPAQ PTS-QAQVAV A-KVN-RPSS

rat PEPEPEPAPE EAAPEVQ--- TLPPEEPVEG EDVAEAGPSL QETQEADPPQ PTS-QAQVAV V-KVN-RPSS

bovine PETAPE---E TELEEV---- SLPPEEPCVG KEVAAVTLGP QGTQETALTP PTSLQAQVSV APEAHSSPRG

* * **** * * **

canine WVLTWLKKSV EKVVPQPVPS SRLAQSTAAG GEGPA-QAGA QVSGQCSTG- --SSDGLGEA --AGDTGSGP

human RVLTWLMKGV EKVIPQPVHS IT-------- -EDP-----A QILGHGSTGD TGCTDEPNEA LEAQDTRPGL

mouse WMLSWFWRGM QKVVPQPV-C SNGGQNLAAG ERDPD-QGGA QIPEPCGTGD PGSAEASG-- --TQDTEPSL

rat WMLSWFWKGM EKVVPQPVYS SSGGQNLAAG EGGPD-QDGA QTLEPCGTGD PGSEDGSDKT SKTQDTEPSL

bovine WVLTWLRKGV EKVVPQPAHS SRPSQNIAAG LESPDQQAGA QILGQCGTG- --GSDEPSEP SRAEDPGPGP

* * ** *** * * * *

canine WLLRWLEQNL EKVLPQPPKT SKDQRDEP-- -ADAALDTEP PGPTLETEPV LQAPESSCVP TAGPLEPQEE

human RLLLWLEQNL ERVLPQPPKS SEVWRDEPAV ATGAASDPAP PGRPQEMGPK LQARETPSLP TPIPLQPKEE

mouse WLLRWLEQNL EKVLPQPPPP SLAWKVEP-- -EAAVLDPDP PGTPMQMEPT ----ESPSQP NPGPLEPEEE

rat WLLRWLELNL EKVLPQPPTP SQAWKVEP-- -EGAVLEPDP PGTPMEVEPT ----ENPSQP NPGPVEPEEE

bovine WLLRWFEQNL EKMLPQPPKI SEGWRDEP-- -TDAALGPEP PGPALEIKPM LQAQESPSLP APGPPEPEEE

** * * ** * ***** * ** * * ** * * * * * **

canine ----PPSEPQ LSPQA--SSL LLPSDPARLM AWLLHRLEMA LPQPVLHGKA REQEPDSPVT CDVQTICILP

human PKEAPAPEPQ PGSQAQTSSL PPTRDPARLV AWVLHRLEMA LPQPVLHGKI GEQEPDSPGI CDVQTISILP

mouse ----PAAEPQ PGFQS--SSL PPPGDPVRLI EWLLHRLEMA LPQPVLHGKA AEQEPGCPGM CDVQTISILP

rat ----PAAEPQ PGFQA--SSL PPPGDPVRLI EWLLHRLEMA LPQPVLHGKA AEQEPSCPGT CDVQTISILP

bovine ----PIPEPQ PTIQA--SSL PPPQDSARLM AWILHRLEMA LPQPVIRGKG GEQESDAPVT CDVQTISILP

* *** * *** * ** * ******* ***** ** *** * ****** ***

canine GGQEEPDLVL EEVDPHWEED EHQDG--GAS PQDSEAAPAY EEENEAVEEM PRKLPWIQEE REDEEEDGEE

human GGQVEPDLVL EEVEPPW-ED AHQDV--STS PQGTEVVPAY EEENKAVEKM PRELSRIEEE KEDEEEEEEE

mouse VEQVEHDLVL EEVDSCWEDA QQEDG---AS PQETEVAPAH EEESEAIVEI PRELTKIQEE REDEQEEDEE

rat VEQAEHDLVL EDVDSCWEDT QQEDG---AS LQETELAPIY EDESEAMVEM PRELPQIQEQ QEEENEEKEE

bovine GEQEESHLIL EEVDPHWEED EHQEGSTSTS PRTSEAAPAD EEKGKVVEQT PRELPRIQEE KEDEEEEKED

* * * * * * * * * * * ** * * * * * *

canine EGED------ ---------- ---------- ---------- ---------- ---------- ----------

human EEEE------ ---------- ---------- ---------- ---------- ---------- ----------

mouse EKEE------ ---------- ---------- -----EKKKG EEEKEKEEEE KEKEKEKEEE KEEEEKEEEE

rat EEEEKEEKEE KEEEEEKEEE EKREEEKKKE KEEEKKEKEE EENGEEEEKE EKEEKEEEEG KEEKEEKEEK

bovine GEEEEEEGRE KEE----EEG EEKEEEEGRE KEEEEGEKKE EEGREKEEEE GGEKEDEEGR EKEEEEGRGK

*

canine ---------- ---------- ---------- EEEANVLLDS CLEAQAGEDL TGVDRSQPQR AS--------

human ---------- ---------- --------EE EEVTEVLLDS CVVSQVGVGQ SEEDGTRPQS TSDQKLWEEV

mouse EEEKEEEEEE KEEEEKEEEE KEEEEEEEEE EEEPIVLLDS CLVVQADVDE CQLERTPSEL AS--------

rat EEKEEEEKEE KEEEEKEEKE EEEEEEEEEE EEEPIVLLDS CLVVQADVDQ CQLERAQPET AS--------

bovine EEEEGGEKEE EEGRGKEEVE GREEEEDEEE EQDHSVLLDS YLVPQSEEDR SEESETQDQS EVGGAQAQGE

* ***** *

canine ---------- ---------- ---------- ---------- ---------- -----QQELQ EEAVTTSPEV

human GEEAKKEAEE KAKEEAEEVA ---------- ---------- -EEEAEKEPQ DWAETKEEPE AEAEAASSGV

mouse ---IQELPEE KEEKEEEK-- ---------- ---------- --EEEKEEEE EKKEEEVEKK EEGEATNSTV

rat ---IQELPEE EEEKEEEKK- ---------- ---------- -EEEEEKEEE EEKEEEEEKE EEGEATNSTV

bovine VGGAQALSEE SETQDQSEVG GAQDQSEVGG AQAQGEVGGA QEQDGVGGAQ DQSTSHQELQ EEALADSSGV

* * *

canine PATKEHPEVQ VEDVDADSHP LIV-ENTPSP ELPPPSPAKA DTLTVPGSAA GTERKRLPSQ DDEAEELRAL

human PATKQHPEVQ VEDTDADSCP LMAEENPPST VLPPPSPAKS DTLIVPSSAS GTHRKKLPSE DDEAEELKAL

mouse PATKEHPELQ VEDTDADSGP LIPEETLPPP ERPPPSPVKS DTLTVPGAAA AGHRKKLPSQ DDEAEELKAL

rat PATKEHPELQ VEDTDAEAGP LIPEETIPPP ERPPVSPAKS DTLAVPSAA- -THRKKLPSQ DDEAEELKAL

bovine PATEEHPELQ VEDADADSRP LIAEENPPSP VQLPLSPAKS DTLAVPGSAT GSLRKRLPSQ DDEAEELKML

*** *** * *** ** * * * * * ** * *** ** * ** *** ******* *

canine SPAESPMVAW SDPSSPQGTD GQDRATSTAS QNSAIINDRL QELVKLFKER TEKVKEKLID PDVTSDEESP

human SPAESPVVAW SDPTTPKDTD GQDRAASTAS TNSAIINDRL QELVKLFKER TEKVKEKLID PDVTSDEESP

mouse SPAESPVVAW SDPTTPQEAD GQDRAASTAS QNSAIINDRL QELVKMFKER TEKVKEKLID PDVTSDEESP

rat SPAESPVVAW SDPTTPQEAD GEDRAASTAS QNSAIINDRL QELVKMFKER TEKVKEKLID PDVTSDEESP

bovine SPAASPVVAW SDPTSPQGTD DQDRATSTAS QNSAIINDRL QELVKLFKER TEKVKEKLID PDVTSDEESP

*** ** *** *** * * *** **** ********* ***** **** ********** **********

canine KPSPAKKAPE PAPVVKPAEV GQAEEEEHYC DMLCCKFKRR PWKTYRFPQS IDPLTNLMYI LWLFFVVLAW

human KPSPAKKAPE PAPDTKPAEA EPV-EEEHYC DMLCCKFKHR PWKKYQFPQS IDPLTNLMYV LWLFFVVMAW

mouse KPSPAKKAPE PDPAQKPAEA EVA-EEEHYC DMLCCKFKRR PLKMYRFPQS IDPLTNLMYI LWLFFVVLAW

rat KPSPAKKAPD SAPAQKPAEA EAA-EEEHYC DMLCCKFKRR PWKMYQFPQS IDPLTNLMYI LWLFFVVLAW

bovine KPSPAKKAPE PAPEVKPAEA GQV-EEEHYC EMLCCKFKRR PWKKYQFPQS IDPLTNLMYI LWLFFVVLAW

********* * **** ****** ******* * * * * **** ********* ******* **

canine NWNCWLIPVR WAFPYQTPNN IHLWLLMDYL CDLIYLLDIT VFQLRLQFVR GGDIITDKKE MRDNYLKSRR

human NWNCWLIPVR WAFPYQTPDN IHHWLLMDYL CDLIYFLDIT VFQTRLQFVR GGDIITDKKD MRNNYLKSRR

mouse NWNCWLIPVR WAFPYQRADN IHFWLLMDYL CDFIYLLDIT VFQMRLQFVK GGDIITDKKE MRNNYLKSRR

rat NWNCWLIPVR WAFPYQRADN IHLWLLMDYL CDFIYLLDIT VFQMRLQFVK GGDIITDKKE MRNNYLKSQR

bovine NWNCWLIPVR WAFPYQTPDN IHLWLLMDYL CDLIYLLDIT VFQMRLQFVR GGDIITDKKE MRNNYVKSQR

********** ****** * ** ******* ** ** **** *** ***** ********* ** ** ** *

canine FKMDMLCLLP LDFLYLKFGV NPLLRLPRCL KYMAFFEFNS RLESILSKAY VYRVIRTTAY LLYSLHVNSC

human FKMDLLSLLP LDFLYLKVGV NPLLRLPRCL KYMAFFEFNS RLESILSKAY VYRVIRTTAY LLYSLHLNSC

mouse FKMDLLCLLP LDFLYLKLGI NPLLRLPRCL KYMAFFEFNN RLEAILSKAY VYRVIRTTAY LLYSLHLNSC

rat FKMDLLCLLP LDFLYLKLGV NPLLRLPRCL KYMAFFEFNN RLEAILSKAY VYRVIRTTAY LLYSLHLNSC

bovine FKMDMLCLLP LDLLYLKFGV NPLLRLPRCL KYMAFFEFNN RLESILSKAY VYRVIRTTAY LLYSLHLNSC

**** * *** ** **** * ********** ********* *** ****** ********** ****** ***

SS YTGL-

canine LYYWASAYQG LGSTHWVYDG VGNSYIRCYY WAVKTLITIG GLPDPRTLFE IVFQGLNYFT GVFAFSVMIG

human LYYWASAYQG LGSTHWVYDG VGNSYIRCYY FAVKTLITIG GLPDPKTLFE IVFQLLNYFT GVFAFSVMIG

mouse LYYWASAFQG IGSTHWVYDG VGNSYIRCYY WAVKTLITIG GLPDPQTLFE IVFQLLNYFT GVFAFSVMIG

rat LYYWASAFQG IGSTHWVYDG VGNSYIRCYY WAVKTLITIG GLPDPQTLFE IVFQLLNYFT GVFAFSVMIG

bovine LYYWASAYEG LGSTHWVYDG VGNSYIRCYY WAVKTLITIG GLPDPRTLFE IVFQGLNYFT GVFAFSVMIG

******* * ********* ********** ********* ***** **** **** ***** **********

canine QMRDVVGAAT AGQTYYRSCM DSTVKYMNFY KIPRSVQNRV KTWYEYTWQS QGMLDESELM VQLPDKMRLD

human QMRDVVGAAT AGQTYYRSCM DSTVKYMNFY KIPKSVQNRV KTWYEYTWHS QGMLDESELM VQLPDKMRLD

mouse QMRDVVGAAT AGQTYYRSCM DSTVKYMNFY KIPRSVQNRV KTWYEYTWHS QGMLDESELM VQLPDKMRLD

rat QMRDVVGAAT AGQTYYRSCM DSTVKYMNFY KIPRSVQNRV KTWYEYTWHS QGMLDESELM VQLPDKMRLD

bovine QMRDVVGAAT AGQTYYRSCM DSTVKYMNFY KIPRSVQNRV KTWYEYTWHS QGMLDESELM VQLPDKMRLD

********** ********** ********** *** ****** ******** * ********** **********

canine LAIDVNYNIV SKVALFQGCD RQLIFDMLKR LRSVVYLPND YVCKKGEIGR EMYIIKAGEV QVLGGPDGKA

human LAIDVNYNIV SKVALFQGCD RQMIFDMLKR LRSVVYLPND YVCKKGEIGR EMYIIQAGQV QVLGGPDGKS

mouse LAIDVNYSIV SKVALFQGCD RQMIFDMLKR LRSVVYLPND YVCKKGEIGR EMYIIQAGQV QVLGGPDGKA

rat LAIDVNYNIV SKVALFQGCD RQMIFDMLKR LRSVVYLPND YVCKKGEIGR EMYIIQAGQV QVLGGPDGKA

bovine LAIDVNYSIV SKVALFQGCD RQMIFDMLKR LRSVVYLPND YVCKKGEIGR EMYIIQAGQV QVLGGPDGKS

******* ** ********** ** ******* ********** ********** ***** ** * *********

↓

canine VLVTLKAGSV FGEISLLAVG GGNRRTANVV AHGFTNLFIL DKKDLNEILV HYPESQKLLR KKARRMLRNN

human VLVTLKAGSV FGEISLLAVG GGNRRTANVV AHGFTNLFIL DKKDLNEILV HYPESQKLLR KKARRMLRSN

mouse VLVTLKAGSV FGEISLLAVG GGNRRTANVV AHGFTNLFIL DKKDLNEILV HYPESQKLLR KKARRMLRNN

rat VLVTLKAGSV FGEISLLAVG GGNRRTANVV AHGFTNLFIL DKKDLNEILV HYPESQKLLR KKARRMLRNN

bovine VLVTLKAGSV FGEISLLAVG GGNRRTANVV AHGFTNLFIL DKKDLNEILV HYPESQKLLR KKARRMLRNN

********** ********** ********** ********** ********** ********** ******** *

canine NKPKEPKSVL ILPPRAGTPK LFNAALAVAG KMGAKGAKAG KLAHLRARLK ELAALEAAAR QQQLLEQAKS

human NKPKEEKSVL ILPPRAGTPK LFNAALAMTG KMGGKGAKGG KLAHLRARLK ELAALEAAAK QQELVEQAKS

mouse NKPKEEKSVL ILPPRAGTPK LFNAALAAAG KMGPRGAKGG KLAHLRARLK ELAALEAAAR QQQLLEQAKS

rat NKPKEEKSVL ILPPRAGTPK LFNAALAAAG KMGPRGAKGG KLAHLRARLK ELAALEAAAR QQQLLEQAKS

bovine NKPKE-KSVL ILPPRAGTPK LFNAALAAAG KMGAKGGRGG RLALLRARLK ELAALEAAAR QQQLLEQAKS

***** **** ********** ******* * *** * * ** ****** ********* ** * *****

3

canine SQDAA-GEAG QAAPDQDQPP APEHPESQEP PATRSSP--- -------PAS PPASP-PAS- ERPEEGGEGE

human SQDVK-GEEG SAAPDQHTHP K-EAATDPPA PRTPPEP--- -------PGS PPSSPPPASL GRPEGEEEGP

mouse SQEAG-GEEG SGATDQPAPQ EPPEPKDPPK PPGPPEP--- -------SA- -QSSPPPAS- AKPEESTGEA

rat SQEAG-GEEG SGATDQPAPQ EPSEPKEP-- ----PEP--- -------PA- -PSSPPPAS- AKPEGSTEEA

bovine SEDAAVGEEG SASPEQPPRP EPPAPEAPAP EPTAPEPLAP EAPAPEAPA- -PSSPPPASQ ERPEGD-KDA

* ** * * ** *** **

canine AGPSEPSVLI RMSPGPDPSE QILSVEVPEE KKEAE----- ---------- -------

human AEPEEHSVRI CMSPGPEPGE QILSVKMPEE REEKAE---- ---------- -------

mouse AGPPEPSVRI RVSPGPDPGE QTLSVEVLEE KKEGAE---- ---------- -------

rat AGPPEPSVRI RVSPGPDPGE QTLSVEMLEE KKEEVE---- ---------- -------

bovine ARPEEHPVRI HVTLGPDPSE QILLVEVPEK QEEKEKKEEE TEEKEEGEEA RKEKEEE

* * * * * ** * * * * * * *
