# Supplementary material for: A Large Animal Model for CNGB1 Autosomal Recessive Retinitis Pigmentosa
Source: PLoS One. 2013 Aug 19;8(8):e72229. doi: 10.1371/journal.pone.0072229 (PMC3747135; doi:10.1371/journal.pone.0072229)
Supplement: Methods S1 — (DOCX) [file pone.0072229.s006.docx]

**Supplemental Methods**

**CNGB1 genotyping assay**


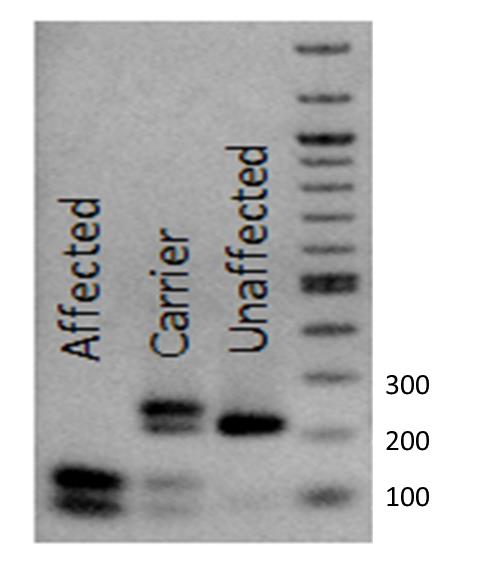
PCR primers (F primer 5’-AAGCGTTTGCATCTTGCAC-3’ and R primer 5’-TGTCCGATCATCACAGAGAAAG-3’; underlined below) were designed to amplify *CNGB1* exon 26 (uppercase text below). PCR amplification produces a 239 bp product in unaffected dogs and a larger 244 bp product in affected dogs (due to the 1 bp deletion and 6 bp insertion; red highlighted text below). The enzyme *AluI,* which recognizes the sequence AGCT, can be used to distinguish the normal and mutant alleles. The PCR product is digested for 2 hr at 37°C with 5 units *AluI* enzyme in 1X NEB buffer 4 (New England Biolabs, Ipswitch, MA). There is a common *AluI* site (blue highlighted text below) in both unaffected and affected dog DNA that verifies restriction enzyme activity. The unaffected dogs have the common 21 bp product (does not appear on gel) and a 218 bp product. The affected dogs, due to the 6 bp insertion (AGCTAC) that contains an *AluI* cut site, produce the 21 bp common band and the diagnostic 95 and 128 bp bands. A heteroduplex band is also seen in carriers, which migrates slightly slower than the 218 bp band. Note that the genotyping assay is designed utilizing the 6 bp insertion, not the 1 bp deletion. It is assumed that these two sequence changes are in complete linkage disequilibrium.

**Unaffected dog Sequence**

aagcgtttgcatcttgcacagctgtcagagccaggccacccaggctcagcagggtggggcctttgctgaccgaggtccccttcctcttctctctctagTTACATTCGCTGTTACT------ACTGGGC TGTGAAGACCCTCATCACCATTGGTGGGCTGCCTGACCCCAGAACGCTCTTTGAAATTGTCTTCCAGGGGCTGAACTATTTCACGGGTGTCTTTGCTTTCTCTGTGATGATCGGACAGgtagctgggtcaccagtctggcttgccacccacccgtctgccagggcccctcccactactgccagaaatgtcaccatctgtggccattccttttggactaagtctgggaaagagcacgagt

**Affected dog sequence**

aagcgtttgcatcttgcacagctgtcagagccaggccacccaggctcagcagggtggggcctttgctgaccgaggtccccttcctcttctctctctagTTACATTCGCTGTT_CTAGCTACACTGGGCTGTGAAGACCCTCATCACCATTGGTGGGCTGCCTGACCCCAGAACGCTCTTTGAAATTGTCTTCCAGGGGCTGAACTATTTCACGGGTGTCTTTGCTTTCTCTGTGATGATCGGACAGgtagctgggtcaccagtctggcttgccacccacccgtctgccagggcccctcccactactgccagaaatgtcaccatctgtggccattccttttggactaagtctgggaaagagcacgagt
